# Distribution patterns and community assembly processes of generalists and specialists in river affected by wastewater discharge from distilleries in typical Chinese spirits production area in China

**DOI:** 10.3389/fmicb.2026.1860769

**Published:** 2026-07-09

**Authors:** Lixi Deng, Ying Huang, Qihong Zhong, Yu Mu, Dong Li, Yaxi Liu, Hong Zhang, Chi Zhao, Shirui Yu, Fu Jiao, Xiuyuan Yang

**Affiliations:** 1 School of Food Engineering, Moutai Institute, Renhuai, China; 2 School of Brewing Engineering, Moutai Institute, Renhuai, China; 3 School of Resources and Environment, Moutai Institute, Renhuai, China; 4 Kweichow Moutai Co. Ltd., Renhuai, China; 5 Guizhou Institute of Biology, Guizhou Academy of Sciences, Guiyang, China; 6 Fanjing Mountain Ecological Station of Guizhou Institute of Biology, Guizhou Academy of Sciences, Tongren, China

**Keywords:** community assembly, distillery wastewater, distribution patterns, environmental adaptability, phylogenetics

## Abstract

**Introduction:**

Understanding how bacterial communities respond to distillery wastewater discharge into rivers is crucial for restoring and maintaining aquatic ecosystem functions. However, feedback mechanisms involving bacterial generalists and specialists under distillery wastewater disturbance remain unexplored.

**Methods:**

This study employs high-throughput sequencing, neutral community models, and null models to analyze, for the first time, the distribution patterns, environmental adaptations, assembly processes, and driving factors of generalists and specialists upstream and downstream of a spirits wastewater discharge site in the Chishui River.

**Results:**

Proteobacteria emerged as the dominant phylum among both specialists and generalists, exhibiting significant changes before and after wastewater discharge. Specialists *α*-diversity showed significant differences along the river flow direction (*p* < 0.05), with specialists generally displaying higher phylogenetic species variability (PSV) and phylogenetic species clustering (PSC) than generalists. Specialists exhibited more complex and compact co-occurrence networks than generalists, and specialist networks demonstrated greater stability. Differences in species formation rates, extinction rates, and diversification rates were observed between generalists and specialists across different river sections. Specialists exhibited stronger environmental adaptability than generalists under high disturbances from spirits wastewater, whereas generalists showed greater resilience under low disturbance conditions. Stochastic processes dominated community assembly, contributing more significantly to the formation of generalists than specialists.

**Conclusion:**

Our findings enhance the understanding of bacterial responses to spirits wastewater and provide valuable insights for the ecological restoration of polluted rivers.

## 1 Introduction

Rivers are vital components of freshwater ecosystems, serving as transport systems for minerals and nutrients, biodiversity hotspots, and key providers of ecological services. Their distribution patterns are jointly influenced by climate and topography (Liu et al., 2021; Liu et al., 2020; Shi et al., 2025b). Human activities contribute to the degradation of river water quality and eutrophication by introducing pollutants and nutrients into water bodies, while also altering river flow and watershed network connectivity (Carles et al., 2022; Ren et al., 2024; Zhao et al., 2025). Changes in water quality not only lead to food web restructuring and disruption of nutrient cycles in river ecosystems but also alter river ecosystem functions and services, potentially causing severe biodiversity loss (de Guzman et al., 2022; Zhang et al., 2019). Riverine microbial communities, including bacteria and fungi, play crucial roles in key ecological processes such as organic matter degradation, participation in biogeochemical cycles, and the maintenance of dynamic equilibrium within ecosystems (Bohórquez-Herrera et al., 2023; Geng et al., 2024; Varjani, 2017). Microorganisms are highly sensitive to changes in water quality and hydrological stress (Yan et al., 2026). Changes in environmental factors such as nutrient availability, oxygen concentration, and light exposure caused by water quality changes play a crucial role in shaping microbial diversity, community assembly, and interaction network structure by affecting organic matter utilization, metabolism, enzyme activity, and the synthesis of proteins and enzymes (Chen et al., 2022; Li et al., 2024; Ma et al., 2025).

Microbial communities typically exhibit highly imbalanced species abundance patterns, characterized by abundant and rare species, with abundant and rare bacteria performing distinct ecological functions within ecosystems (Shi et al., 2023). Bacterial generalists (i.e., those that are able to adapt to diverse habitats) and specialists (i.e., those that adapted to specific habitats) occupy relatively broad and narrow niches, respectively, and perform vital ecological roles in soil and aquatic environments (Al et al., 2022; Wong et al., 2023). Generalist communities occupy a wider range of ecological niches and are adapted to variable environments, whereas specialist communities occupy a narrower ecological niche, are sensitive to environmental changes, and often perform key ecological functions, with both groups playing important ecological roles in terrestrial and aquatic ecosystems and serving as indicator species reflecting environmental changes in aquatic ecosystems (Liu et al., 2025; Yang Q. et al., 2023). The widespread distribution of generalists contributes significantly to ecosystem stability, whereas specialists are sensitive to pollution levels and can respond rapidly to varying environmental conditions (Al et al., 2022; Chen et al., 2019; Gad et al., 2020). Distribution patterns and assembly processes of generalist and specialist communities under natural conditions have been extensively documented in ecosystems such as mountainous (Zhang et al., 2024), lakes (Yan et al., 2022), and agricultural fields (Xu et al., 2022b). Research has also examined the distribution patterns and assembly processes of these communities in aquatic ecosystems under disturbed conditions (Al et al., 2022; Yang Y. et al., 2023), revealing landscape patterns and ecological processes of generalist and specialist communities in water bodies affected by dredging and urban sewage discharge. However, limited research has addressed the landscape characteristics of bacterial generalists and specialists, the generation of phylogenetic diversity, and the ecological mechanisms underpinning ecological processes and ecosystem maintenance in environments disturbed by distillery wastewater.

Chinese spirits industry holds a significant position in the global spirits sector and is currently experiencing a growth trend. While the spirits industry has driven tourism and rural revitalization in multiple countries, the pollution caused by brewing wastewater generated during its production process cannot be overlooked (Latessa et al., 2023). Early studies indicate that discharging distillery wastewater into water bodies adversely affects fish survival, spawning, salmon fly-feeding, and other aquatic organisms, rendering rivers unsuitable for primary uses (Stevenson, 1898; Garcia et al., 2017; Mekonnen and Tekeba, 2024). China ranks among the world’s most renowned producers of baijiu, with the Chishui River celebrated as a “wine river” (Liu et al., 2023). Simultaneously, the Chishui River serves as a vital ecological barrier in the upper Yangtze River basin. Although numerous studies have documented microbial community distribution patterns and assembly processes in the Chishui River (Wu et al., 2022, 2023; Lü et al., 2021), no research has yet examined the effects of distillery wastewater discharge on microbial species differentiation, community assembly, and ecological processes in the Chishui River. This gap prompted our investigation into how distillery wastewater discharge impacts bacterial generalists and specialists.

**Figure 1:**
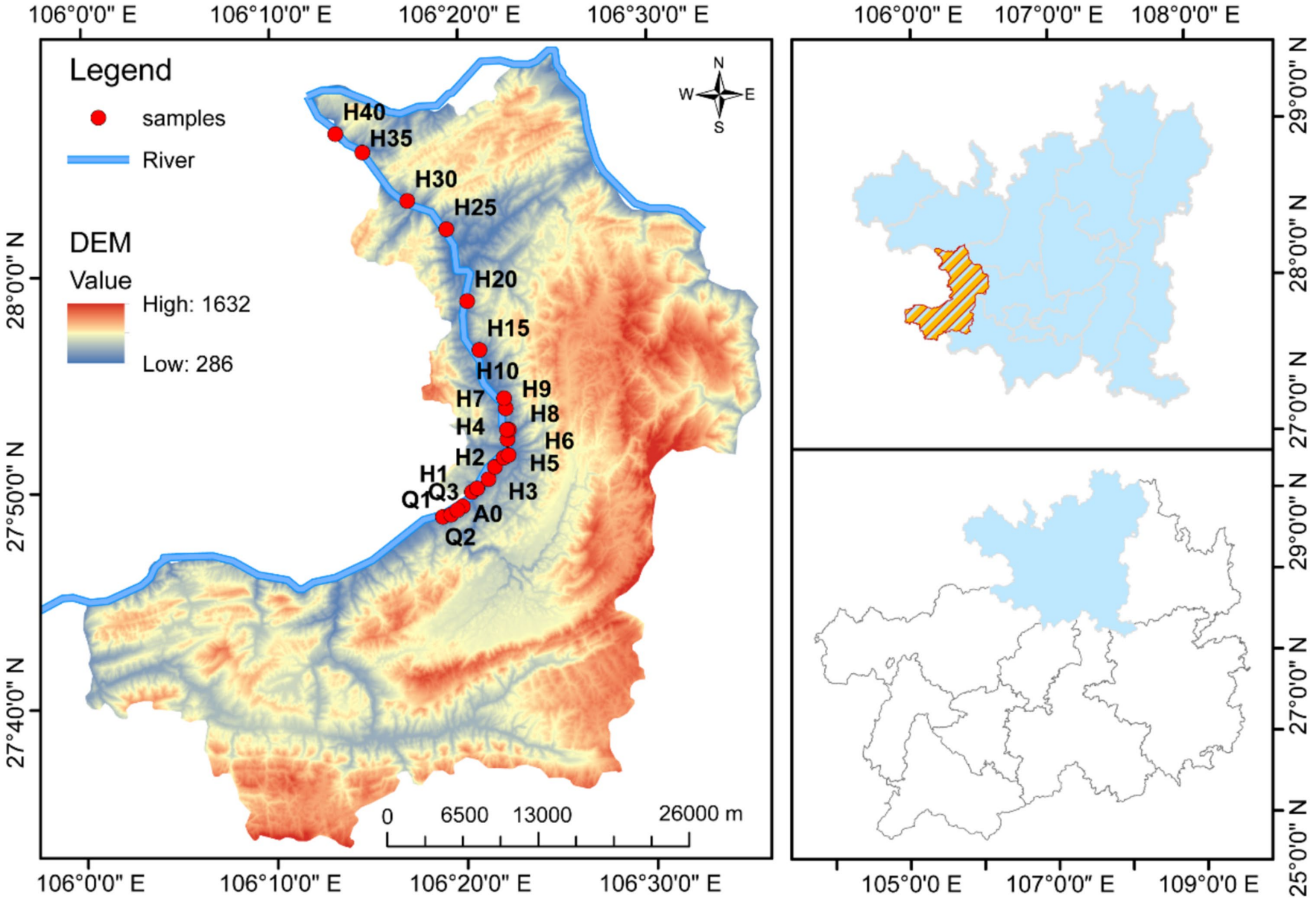
Geographic information of sampling points.

To address this knowledge gap, this study focused on the discharge point of a distillery into the Chishui River basin, as well as various upstream and downstream river segments, as the research area. Sampling sites were selected at different distances from the distillery discharge point to represent the diverse environmental conditions of the affected Chishui River segment (Figure 1). We hypothesized that the discharge of distillery wastewater modified the spatiotemporal distribution of bacterial generalists and specialists, their community assembly processes, and evolutionary potential. This resulted in persistent community differences between generalists and specialists, with both bacterial groups being significantly influenced by environmental factors. The objectives of this study are to (i) explore how distillery wastewater discharge alters the distribution patterns and community assembly of bacterial generalists and specialists; (ii) compare the ecological responses of generalists and specialists’ communities to wastewater disturbance; and (iii) determine the key environmental drivers shaping the dynamics of these microbial groups. The findings are expected to elucidate the principal factors affecting microbial communities following spirits wastewater discharge into riverine systems, thereby providing a scientific foundation for understanding microbial response mechanisms in polluted river ecosystems. Furthermore, this research offers critical insights and support for optimizing ecological assessment and remediation strategies for contaminated aquatic environments.

## 2 Materials and methods

### 2.1 Study area and sampling

The Chishui River constitutes a significant tributary on the southern bank of the upper Yangtze River, with its watershed situated at the confluence of Yunnan, Guizhou, and Sichuan provinces (104°44′–106°59′E, 27°13′–28°50′N). Within Guizhou Province, the watershed encompasses an area of 11448.3 km^2^, representing 60.2% of the total basin area. The climate of the Chishui River basin exhibits notable regional variability. Annual precipitation typically ranges from 700 to 1,100 mm, predominantly occurring between June and September. The basin experiences a continental climate, primarily within the mid-subtropical zone, characterized by dry, cold winters and hot, humid summers. Recorded temperature extremes include a maximum of 39 °C and a minimum of −5 °C, with average temperatures ranging from 15 °C to 20 °C. The Chishui River basin is distinguished for its prosperous wine industry and is recognized as China’s foremost “River of Fine Wine.” Local communities along the river have a longstanding tradition of liquor production. However, the discharge of brewing wastewater has adversely affected the river’s water quality. To evaluate this impact, water samples were collected from the river both upstream (Q1–Q3) and downstream (H1–H40) of a distillery discharge point (A0) (Figure 1). There was a total of 20 sampling points. The 20 sampling sites were categorized into six groups based on their location relative to the discharge point: upstream DTQ (3 km, 3 sites: Q1–Q3), the discharge point DT (1 site), and four downstream groups (H1–H40) at 5 km (DTHA, 5 sites), 10 km (DTHB, 5 sites), 20 km (DTHC, 4 sites), and 40 km (DTHD, 2 sites) (The division of sampling points can be found in section 1.1 of the Supplementary materials). During the June 2024, approximately 1.5 liters of water were collected at each site from depths of 0 to 20 cm. Prior to sampling, containers were rinsed 4–5 times with river water, and samples were filtered using 63 μm nylon filters. In each period, water samples were collected in triplicate at each of the 20 locations, yielding a total of 60 samples per period. Following collection, samples were promptly transported to the laboratory for processing. Some samples were filtered and immediately frozen before being transported under cold chain conditions to BioMarker (Beijing) for DNA extraction and high-throughput sequencing. Additionally, nine wastewater samples were collected from the distillery’s discharge. Detailed results are provided in Supplementary Table S1.

### 2.2 Determination of the physicochemical properties of aquatic environments

The water quality parameters assessed in this study encompassed turbidity (TU), dissolved oxygen (DO), electrical conductivity (EC), pH, total dissolved solids (TDS), salinity (SAL), oxidation–reduction potential (ORP), permanganate-based chemical oxygen demand (COD_Mn_), ammonia nitrogen (NH_3_-N), total nitrogen (TN), total phosphorus (TP), Fe, Al, Ni, and As. Among these, DO, TU, pH, ORP, EC, SAL, and TDS were measured *in situ* using a multi-parameter water quality meter (ProDss, Xylem, USA). TN was quantified employing the alkaline potassium persulfate UV spectrophotometric method, whereas TP was determined via the ammonium molybdate spectrophotometric method (Yang Y. et al., 2023). COD_Mn_ was analyzed in accordance with the procedures outlined in the Methods for Monitoring and Analysis of Water and Wastewater (State Environmental Protection Administration, 2002). Concentrations of Fe, Al, Ni, and As were measured by inductively coupled plasma mass spectrometry (ICP-MS) following filtration of water samples and acidification of the filtrate with concentrated nitric acid (Le and Nguyen, 2024).

### 2.3 Microbial DNA extraction and high-throughput sequencing

Total DNA was extracted from filter membranes utilizing the FastDNA® SPIN Kit in accordance with the manufacturer’s protocol. The V3-V4 region was amplified via PCR employing primers 338F (ACTCCTACGGGAGGCAGCA) and 806R (GGACTACHVGGGTWTCTAAT). The resulting amplification products were purified using Hieff NGS DNA Select Beads (Yeasen Biotechnology Co., Ltd., Shanghai, China). Subsequently, the purified amplicons were sequenced on the Illumina Novaseq PE250 platform (Illumina, USA) at Shanghai Paisen Bio-Tech Co., Ltd. (Shanghai, China). Detailed methodological procedures are described in previous studies (Li et al., 2023; Yang et al., 2025).

### 2.4 Statistical analysis

Quality control and filtering of raw paired-end sequencing reads were conducted using fastp (version 0.19.6). Primer sequences were subsequently removed with cutadapt (version 1.9.1). The DADA2 algorithm implemented within the QIIME2 platform was utilized to denoise sequences, assemble paired-end reads, and eliminate chimeric sequences, resulting in the generation of amplicon sequence variants (ASVs). Following this, all annotated chloroplast and mitochondrial sequences were excluded from the dataset. Taxonomic annotation was performed using the SILVA database (version 138) with a confidence threshold set at 80%. Community composition was then quantified at multiple taxonomic levels for each sample. The detailed analytical procedures have been described in previous studies (Yang Q. et al., 2023; Chen et al., 2025).

Niche breadth was calculated using the Levins method, and the occurrence frequencies of amplicon sequence variants (ASVs) were randomized through permutation (1,000 iterations) to generate the null distribution of ASV niche breadth indices (Levins, 1968; Yang Y. et al., 2023; Zhang et al., 2024). ASVs were classified as generalists or specialists if their observed niche breadth values exceeded or fell below the 95% confidence interval of the null distribution, respectively. ASVs that did not meet these criteria were categorized as “Neutral” taxa (Zhang et al., 2024).

When the data satisfy the assumptions of normality and homogeneity of variance, one-way ANOVA is employed to assess differences among data sets; otherwise, the Kruskal-Wallis and Wilcoxon rank-sum tests are applied. Ordinary least squares regression was utilized to model the relationships between physicochemical properties, microbial diversity, and river distance. Systematic analyses of *α* diversity, specifically the Shannon diversity index, were conducted using the vegan package, while *β* diversity was evaluated through non-metric multidimensional scaling (NMDS) based on Bray-Curtis distances (Yang Q. et al., 2023). Concurrently, phylogenetic diversity metrics (PD, PSV, PSC, and PSR) were computed using the pd. and psd functions within the “picante” R package (Ma et al., 2022). The influence of increasing environmental distance on microbial community similarity was investigated via distance decay analysis. Furthermore, by decomposing microbial community β-diversity, the contributions of species turnover and richness variation to compositional changes were examined (Wan et al., 2023; Yang Y. et al., 2023).

The evolutionary potential of bacterial generalists and specialists was evaluated by estimating speciation, extinction, diversification, and conversion rates (Xu et al., 2022a; Yang Y. et al., 2023). Phylogenetic signals, which indicate the conservation of species functional traits across phylogenies, serve as predictors of microbial evolutionary adaptations. Blomberg’s K statistic was employed to quantify phylogenetic signals within planktonic bacterial groups, with higher K values denoting stronger phylogenetic conservatism (Blomberg et al., 2003; Goberna and Verdú, 2016; Wan et al., 2021). Co-occurrence patterns were constructed using Pearson correlation coefficients, and the Random Matrix Theory (RMT) method was applied to determine the correlation cutoff threshold (r > 0.6, *p* < 0.05) automatically (Yuan et al., 2021). The resulting co-occurrence network was visualized using Gephi (version 0.10.1). Neutrality models were utilized to assess the relative influence of stochastic and deterministic processes on microbial community structure. To further elucidate the contributions of these processes to community assembly, phylogenetic signals were first examined via phylogenetic Mantel correlation plots (Supplementary Figure S2). Subsequently, community assembly was analyzed within the framework proposed by iCAMP and Stegen et al. (2013) [Supplementary materials (section 1.2)]. Sampling locations were mapped using ArcMap 10.8.5. Additionally, relationships between various riverine environmental factors and generalists/specialists were investigated through Mantel tests, correlation analyses, and interpretable machine learning models (XGBoost).

## 3 Results

### 3.1 Characteristics of changes in water indicators along rivers

**Figure 2:**
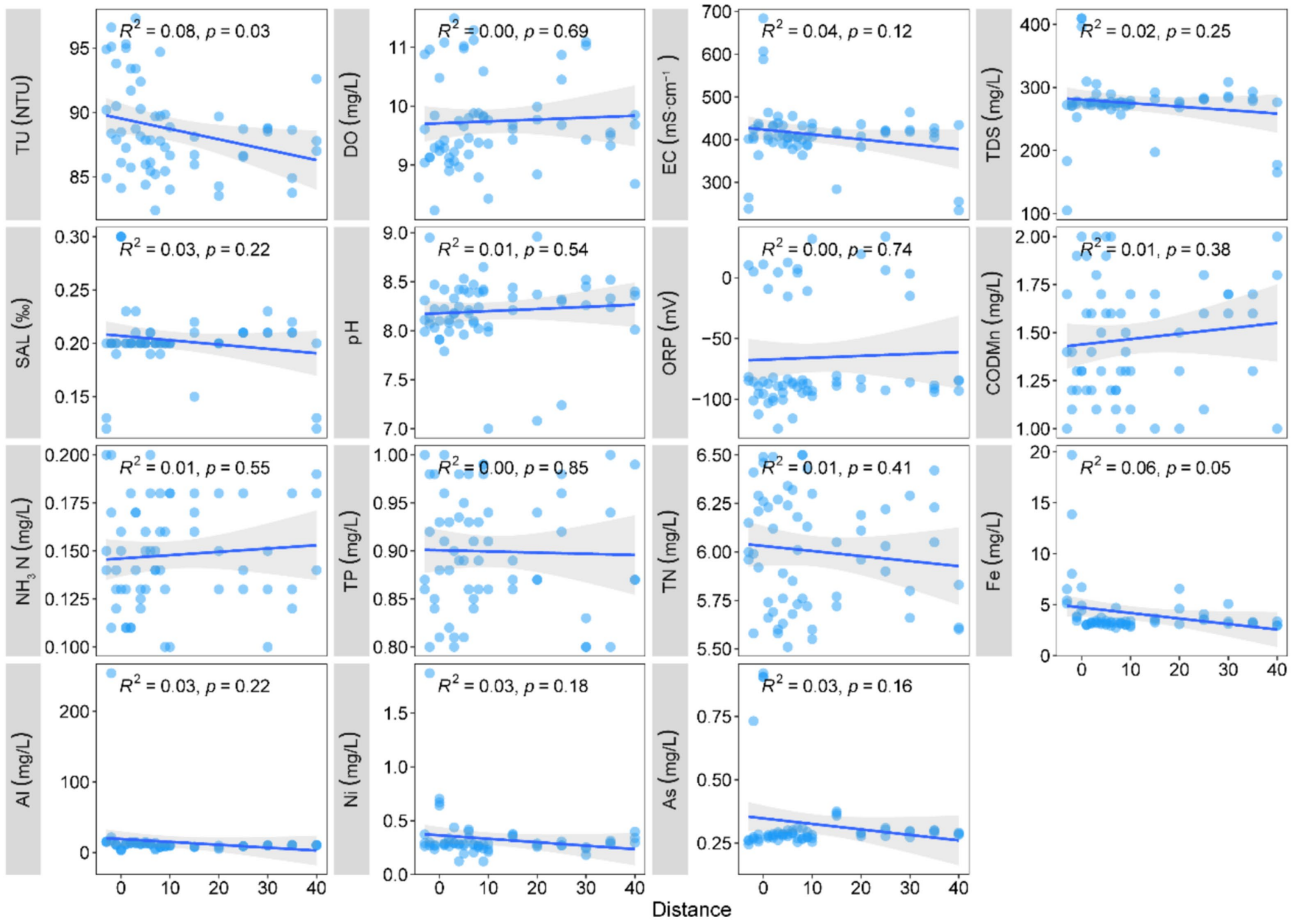
Changes in water indicators along the river flow.

The physicochemical parameters, including TU, EC, TDS, SAL, DO, pH, ORP, COD_Mn_, NH_3_-N, TP, TN, Fe, Al, Ni, and As, showed changes along the river flow direction as shown in Figure 2. Except for TU, which exhibited a significant decreasing trend (*p* < 0.05), there were no significant changes in other physicochemical parameters. We used nonparametric tests to analyze the differences in physicochemical properties among the DTQ, DT, and DTHA-DTHD river sections (Supplementary Figure S2). At different distances from drain outlet of the Chinese Baijiu Distillery, TU, DO, pH, ORP, COD_Mn_, NH_3_-N, TP, and TN did not show significant changes, while EC, TDS, SAL, Fe, Al, Ni, and As exhibited significant differences (*p* < 0.05) in river water at different distances from the discharge outlet. The EC, TDS, and SAL of the river at the discharge outlet (DT) were significantly higher than those of the river water upstream (DTQ) and downstream (DTHA-DTHD) of the discharge outlet. The ion concentrations (Fe, Al, Ni, As) of the water at DT were significantly higher than those of the river water downstream of the discharge outlet. The higher levels of certain physicochemical parameters in the water at DT may be influenced by the distillery’s drainage (Supplementary Table S1).

### 3.2 The division and composition of generalist and specialist communities

**Figure 3:**
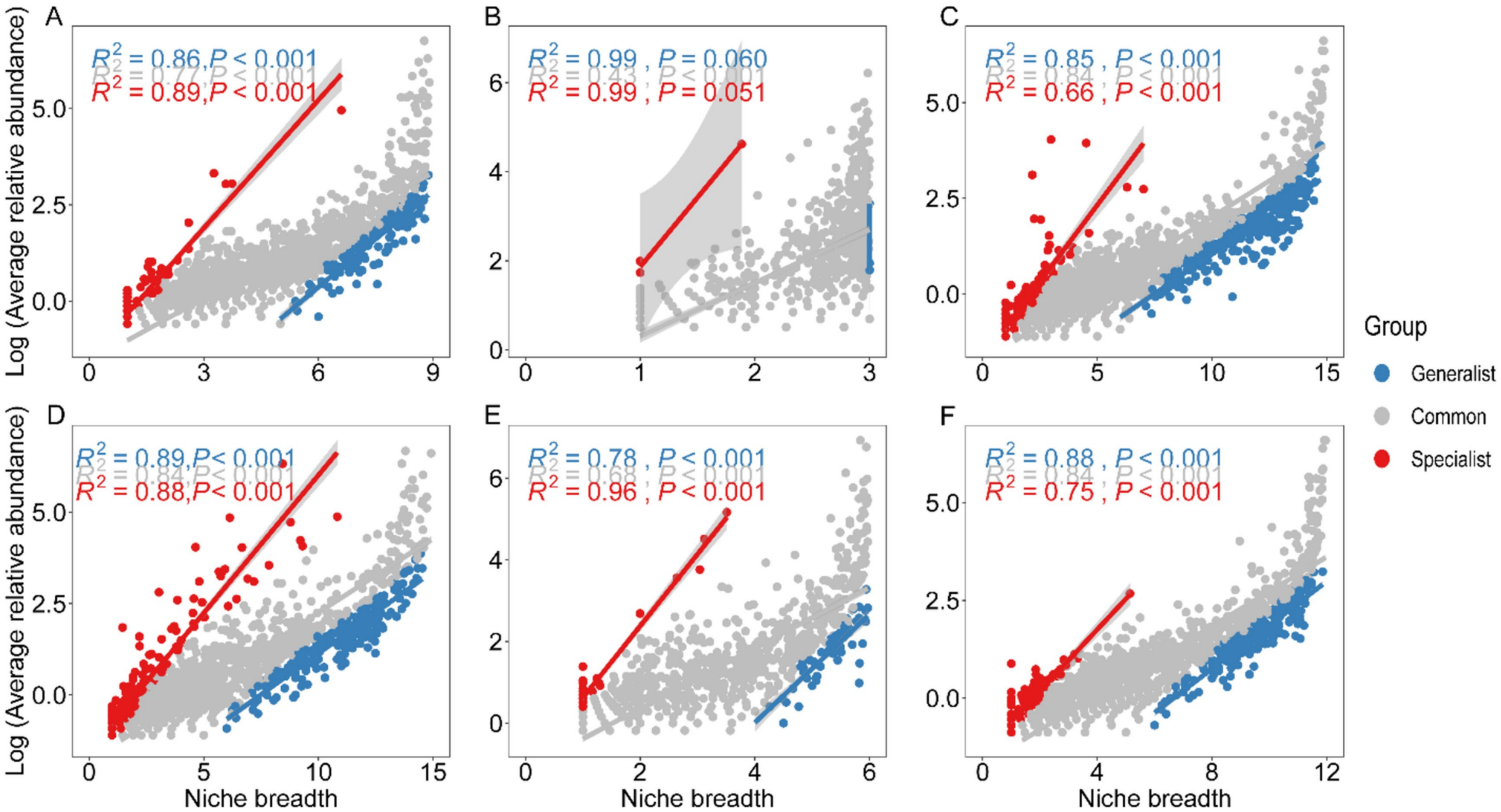
Identification and classification of generalist and specialist categories. **(A)** Represents DTQ; **(B)** represents DT; **(C)** represents DTHA; **(D)** represents DTHB; **(E)** represents DTHC; **(F)** represents DTHD. Due to the impact of distillery wastewater discharge, differentiated microbial communities failed to form in the water body at DT. No longer included in subsequent analysis.

Upstream of the discharge point, 1,503 ASVs were identified, comprising 126 generalist ASVs (8.38%) and 82 specialist ASVs (5.46%). At the discharge point (DT), the persistence or establishment of both specialist and generalist was inhibited (Figure 3B). Conversely, downstream of the discharge point, the relative abundance of specialist and generalist increased markedly, with generalists and specialists accounting for 15.17 and 8.33% at DTHA, 12.81 and 11.37% at DTHB, 5.73 and 4.09% at DTHC, and 10.72 and 7.80% at DTHD, respectively. To further elucidate the distinctions between specialist and generalist, we examined their compositional patterns and observed significant differences in microbial community structure at varying distances upstream and downstream of the distillery effluent outlet. Proteobacteria emerged as the dominant phylum among both specialist and generalist species, with its relative abundance exhibiting pronounced spatial variation along the river (Supplementary Figures S3, S4). Additionally, generalists demonstrated broader niche breadths compared to specialists; however, the niche breadths of both generalist and specialist were significantly diminished at the discharge point (DT) (Supplementary Figure S5).

### 3.3 Biogeographic analysis of generalist and specialist communities

**Figure 4:**
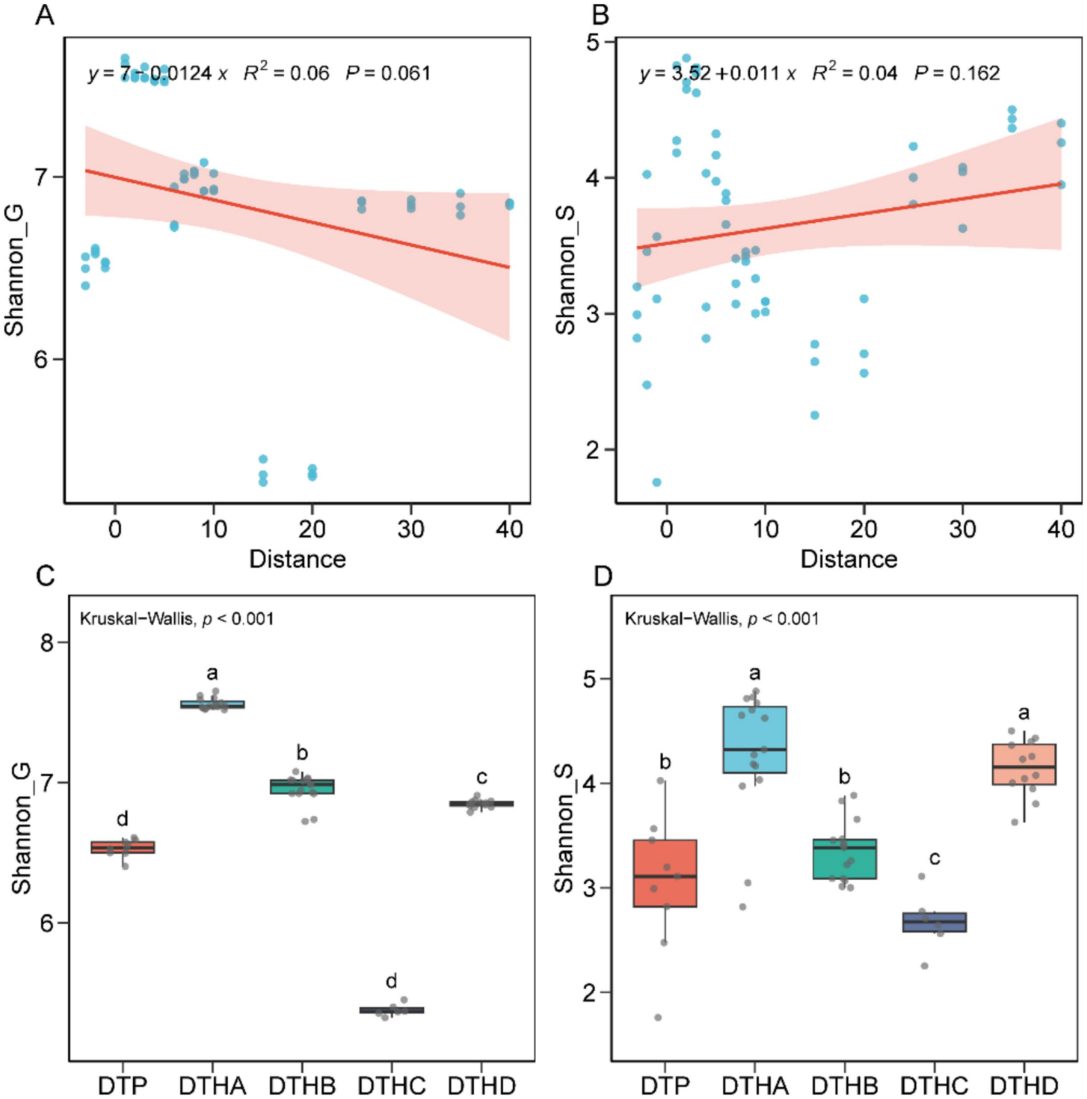
Shannon diversity of generalist and specialist communities. Shannon diversity of generalists **(A,C)** and specialists **(B,D)** along the river flow direction and across different reaches.

Alpha diversity analysis (Figure 4) revealed that specialist communities along the Chishui River exhibited differences in diversity along the river flow direction, whereas generalist communities showed no significant differences. The Shannon diversity of both generalist and specialist communities in the DTHA river section was significantly higher than in other sections. The PD and PSR phylogenetic diversity indices for both generalist and specialist communities displayed similar patterns (Supplementary Figure S6). When using phylogenetic diversity indices independent of richness, the PSV and PSC values of specialists were generally higher than those of generalists. Additionally, PSV and PSC values were generally higher in river segments downstream of the discharge point compared to upstream, with the highest values observed at the discharge point. NMDS plots based on Bray-Curtis distances revealed distinct community compositions among different river segments (Supplementary Figure S7). Analysis of the distance-decay relationship of microbial communities along the Chishui River (Supplementary Figure S8) showed that the correlation between generalist communities and environmental distance was significantly stronger than that between specialist communities and environmental distance. NH_3_-N had the greatest impact on generalist communities, while SAL had the greatest impact on specialist communities, which also exhibited the highest species turnover rate. The steepest slopes corresponding to changes in NH_3_-N and SAL values provide clear evidence of these effects.

### 3.4 Co-occurrence patterns of generalist and specialist communities

**Figure 5:**
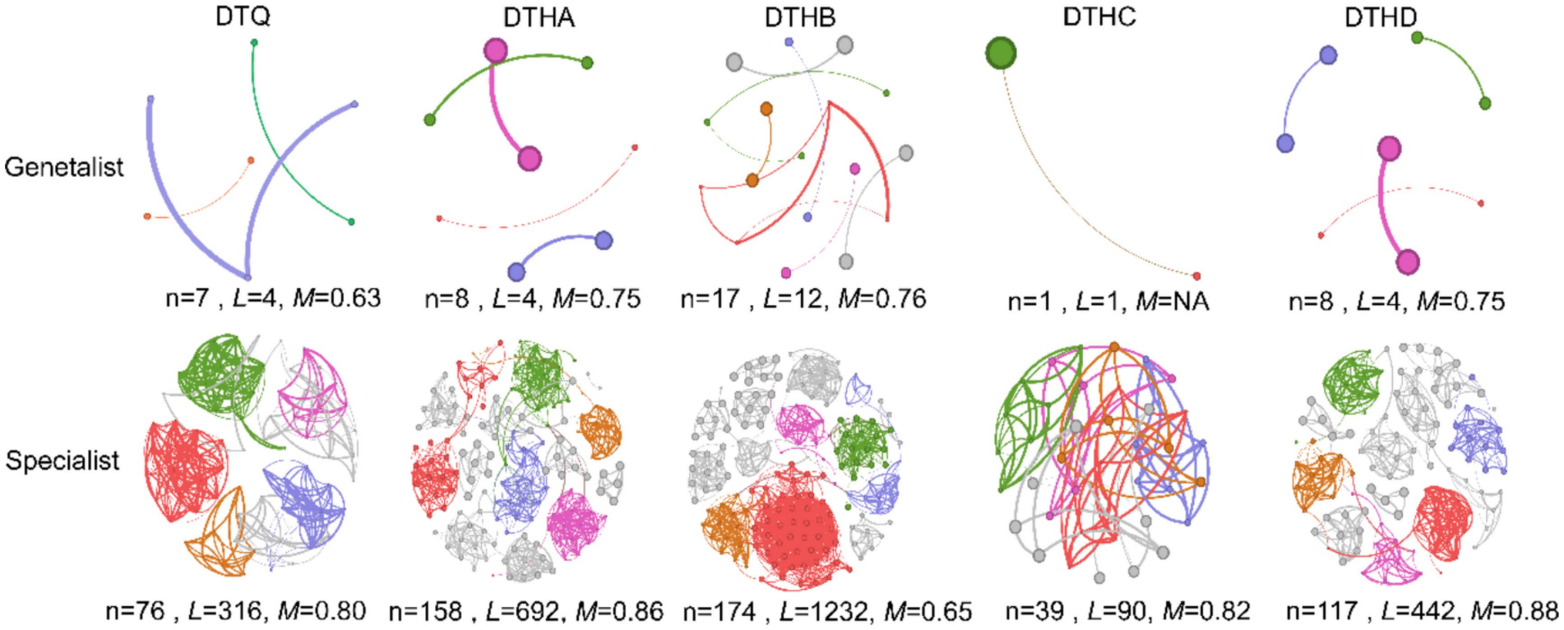
Co-occurrence network analysis of generalists and specialists.

In each river segment at varying distances from the distillery’s drainage, amplicon sequence variants (ASVs) with relative abundance values ≥ 0.01% were selected to construct symbiotic networks representing generalist and specialist communities. A suite of network-level and node-level topological features was computed. Specialists demonstrated higher values in network topology parameters compared to generalists, including greater numbers of nodes and edges, increased modularity, higher average degree, and enhanced graph density (Figure 5; Supplementary Table S2), with significant differences observed among the different river segments. Notably, these topological parameters peaked at the DTHB site relative to other segments. In the co-occurrence networks of generalists, the number of edges exhibiting negative correlations surpassed those with positive correlations (Supplementary Table S2), whereas in the specialists’ networks, all correlations were positive. Collectively, these topological metrics suggested that specialist communities form more complex and cohesive networks than generalist communities. Further analyses involved the removal of specific nodes to evaluate the impact on the natural connectivity of the residual networks (Supplementary Figures S9A–E). Additionally, network robustness was assessed by calculating the proportion of species remaining after the random removal of 50% of species from each actual molecular ecological network (Supplementary Figure S9F). The findings indicated that specialist networks exhibited greater stability and displayed variable trends across different river segments.

### 3.5 The evolutionary potential and environmental adaptability of generalists and specialists

**Figure 6:**
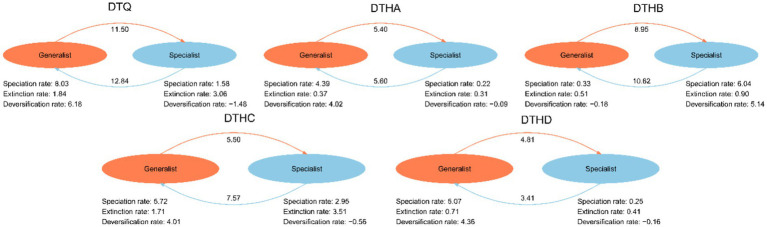
Evolutionary characteristics of generalists and specialists in different river segments upstream and downstream of wastewater discharge point of a distillery. DTQ denoted the segment upstream of wastewater discharge point of a distillery; DTHA-DTHD denoted the segment downstream of wastewater discharge point of a distillery.

The turnover rates from generalist to specialist and from specialist to generalist in the river segment upstream of the drainage outlet (DTQ) were significantly higher than those observed downstream of the drainage outlet (DTHA-DTHD) (Figure 6). Except for the DTHD segment, the turnover rate from specialist to generalist exceeded that from generalist to specialist. The formation, extinction, and diversification rates of generalist species decreased downstream of the distillery drainage outlet, whereas these specialization-related evolutionary traits increased downstream. In the DTHB-DTHC section, the formation and extinction rates of specialists were higher than those of generalists; conversely, in other river sections (DTQ, DTHA and DTHD), the formation, extinction, and diversification rates of generalists surpassed those of specialists. These findings suggested that the evolutionary potential between generalists and specialists differed between upstream and downstream sections. Furthermore, generalists exhibited stronger phylogenetic signals for over 57% of the tested physicochemical factors in DTQ and DTHB-DTHC (Supplementary Figure S10), whereas specialists showed stronger phylogenetic signals for over 70% of these factors in DTHA and DTHD. These results indicated that generalists demonstrated greater environmental adaptability than specialists under conditions of weak external influence, while specialists exhibited stronger adaptability when external factors exert a strong influence. The discharge of distillery wastewater and the subsequent water self-purification process reversed the environmental adaptability differences between generalists and specialists.

### 3.6 The assembly process of a community of generalists and specialists

**Figure 7:**
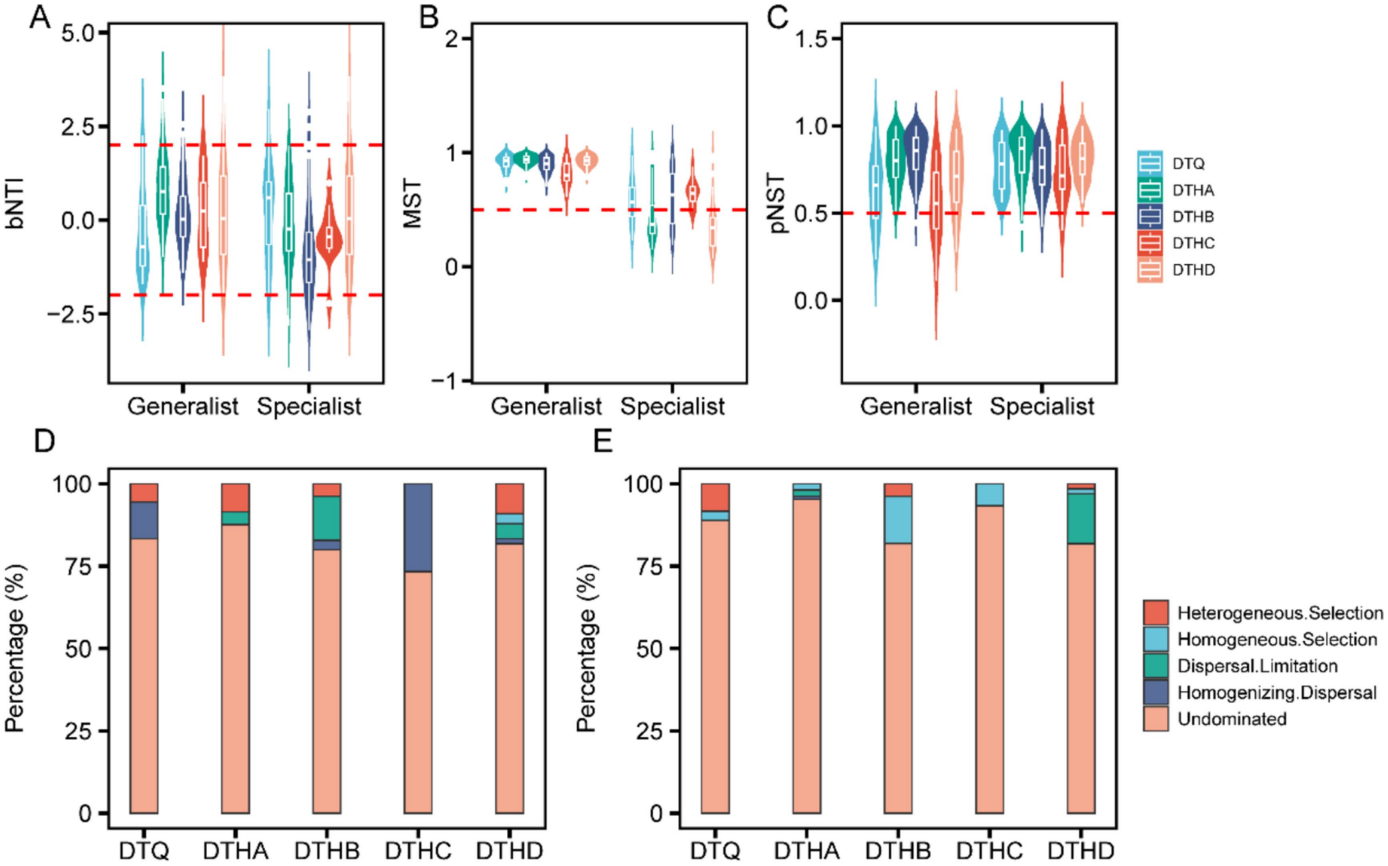
Illustrates the assembly processes of generalist and specialist groups. **(A)** Presented the *β* nearest taxon index (βNTI), where values greater than 2 indicate heterogeneous selection driven by deterministic processes, values less than −2 indicate homogeneous selection by deterministic processes, and values between −2 and 2 suggest the predominance of stochastic processes. **(B)** Showed the Modified Stochasticity Ratio (MST) for different groups based on Bray-Curtis distance; higher MST values denote greater randomness in community assembly. **(C)** Depicted the phylogenetic normalized stochasticity ratio (pNST), with pNST values below 0.5 indicating dominance of deterministic processes and values above 0.5 indicating dominance of stochastic processes. The red dashed line marks the threshold of 0.5 for MST and pNST. **(D)** Detailed the assembly processes of generalist groups as determined by a null model-based framework. **(E)** Detailed the assembly processes of specialist groups as determined by a null model-based framework. Calculate the percentage of detailed ecological processes assigned to random or deterministic processes.

Null-model-based analyses were conducted to calculate the *β* nearest taxon index (βNTI) and phylogenetically normalized stochasticity ratios (pNST) (Figures 7A,C). These analyses indicated that stochastic processes predominantly influenced both generalist and specialist communities across different reaches, as demonstrated by the modified stochasticity ratios (MST) (Figure 7B). To further elucidate the relative contributions of stochastic and deterministic processes, a null model incorporating βNTI and RC_bray_ metrics was employed. The results revealed that stochastic processes contributed substantially more than deterministic processes in all reaches, except for DT, for both generalists and specialists (Figures 7D,E). RC_bray_ analysis identified drift as the principal process governing microbial community assembly in both groups. Additionally, community assembly processes were examined using a neutral model, yielding R^2^ values ranging from 0.49 to 0.66 for generalists and 0.51 to 0.71 for specialists, with taxa falling outside the confidence bounds indicated by the dashed line (Supplementary Figure S11). Generalists exhibited higher migration rates compared to specialists, and the influence of stochastic processes on the assembly of generalists was more pronounced, corroborating the findings from the null model analysis. Correlation analysis between βNTI and environmental variables demonstrated that only DO (r = 0.29, *p* < 0.05) was significantly correlated (Supplementary Table S3).

### 3.7 The relationship between generalist and specialist communities and environmental factors

We investigated the relationship between generalist and specialist communities and environmental factors in rivers. Generalist communities, generalist community diversity, and specialist community diversity were significantly associated with EC, TDS, SAL, and As (*p* < 0.05) (Supplementary Figure S12A), while specialist communities showed no significant association with environmental factors. The dominant species composition of generalist communities was more significantly influenced by river environmental factors (e.g., As, Ni, Fe, SAL, TDS, EC, and turbidity). Except for Proteobacteria, the other three generalist phyla species showed significant negative correlations with As, Ni, Fe, SAL, TDS, EC, and turbidity. Interestingly, the dominant species composition of specialist communities was less influenced by environmental factors and primarily affected by the distance from the distillery discharge outlet. Machine learning (Supplementary Figures S12D–K) and Monte Carlo analysis further confirmed that Distance, Fe, and Al were the primary influencing factors for generalist communities, while Distance and Ni were the primary influencing factors for specialist communities.

## 4 Discussion

### 4.1 Impacts of distillery effluent on generalist and specialist riverine landscapes

Although the distillery’s drainage was treated prior to its discharge into the river, it continued to impose fundamental environmental constraints on the aquatic ecosystem, such as elevated levels of suspended solids and acidic substances, which lead to notable alterations in bacterial community. This study demonstrated that the wastewater discharge from spirits distilleries into the river significantly altered the physicochemical properties of the river. Specifically, EC, TDS, SAL, and concentrations of ions such as Fe, Al, Ni, and As were markedly elevated at the discharge point (DT) compared to both the pre-discharge (DTQ) and post-discharge points (DTHA–DTHD). These findings were consistent with those of Ipeaiyeda and Onianwa (2009), who reported that spirits wastewater inflow substantially increased levels of chloride, nitrate, ammonia, dissolved solids, turbidity, and biochemical oxygen demand, all of which contributed to the deterioration of river water quality. Furthermore, domestic waste and agricultural pollutants have also been identified as significant factors influencing river water quality (Zhu et al., 2024). Atmospheric precipitation, as well as the erosion of abandoned mines by atmospheric precipitation and shallow groundwater to form coal gangue water flowing into rivers, are potential important factors that may lead to an increase in heavy metal content. Alterations in the physicochemical characteristics of river water subsequently impacted the diversity, composition, and distribution patterns of microbial communities. However, the effects of wastewater discharge from spirits distilleries on riverine generalists and specialists remained insufficiently characterized, particularly within the karstic Chishui River basin. This study revealed that generalists were more abundant than specialists and occupied broader ecological niches across different river reaches, whereas specialists exhibited greater diversity. This observation aligns with previous research (Mo et al., 2021; Yang Y. et al., 2023) and may reflect inherent differences in the adaptive strategies of generalists and specialists to environmental conditions. Proteobacteria constituted the dominant phylum among both generalists and specialists; notably, specialists exhibited a higher relative abundance of Proteobacteria than generalists, a finding that contrasts with earlier studies (Luo et al., 2019; Yang Y. et al., 2023). Such discrepancies might be attributed to geographic and environmental heterogeneity, which influence the distribution patterns, diversity, and community composition of generalists and specialists across sites (see Figures 3, 4C,D, 5; Supplementary Figures S3, S4). Environmental parameters, including temperature and dissolved oxygen, were critical for the growth and development of riverine bacterial communities. Inputs of drainage water, such as sewage and spirits effluent, significantly affect community diversity, composition, and structure (Shi et al., 2025a; Zhang et al., 2020; Zinger et al., 2012). Prior studies have demonstrated that sewage discharge introduces high pollutant loads into rivers, resulting in reduced diversity and abundance of microbial communities (Ren et al., 2024; Xie et al., 2022). Although the spirits effluent discharged into the Chishui River underwent treatment, it nonetheless exerted a substantial impact on the microbial community at the discharge site, inducing significant alterations in community structure, composition, diversity, and subspecies differentiation (Figures 3, 4C,D; Supplementary Figures S3, S4). For instance, the formation of generalists and specialists was notably disrupted in the water column at the effluent entry point. Conversely, the negative impact on microbial diversity and composition decreases with increasing distance from the discharge site, likely due to the reduction of alcohols, organic matter, and other contaminants via natural self-purification processes, as well as the resilience and resistance of generalists and specialists to environmental fluctuations (Comont et al., 2020; Yang Y. et al., 2023). It is worth noting that seasonal changes (such as wet and dry seasons) could cause drastic changes in environmental factors such as flow and water temperature, which undoubtedly profoundly affected the self-purification process of rivers and thus have an impact on the survival of microorganisms. Additionally, co-occurrence network analyses revealed predominantly negative correlations among generalists, whereas specialists exhibited positive interspecific correlations, with the specialist network demonstrating greater stability. This pattern may reflect the tendency for species within external pressure, such as those impacted by wastewater inputs, to form positive associations in response to environmental stressors (Yang Q. et al., 2023). In summary, wastewater discharge from spirits distilleries significantly altered the distribution patterns and community structures of microbial generalists and specialists within the river ecosystem.

### 4.2 Variations in evolutionary potential and environmental adaptability between generalist and specialist influenced by wastewater discharge from spirits distilleries

In this study, we used the BiSSE model and phylogenetic signals to estimate and infer the evolutionary characteristics of generalists and specialists in river microenvironments. This study demonstrated that spirits drainage, as a significant anthropogenic disturbance, substantially altered the evolutionary potential and environmental adaptation patterns of both generalists and specialists. Prior to drainage, the reversal rate between generalists and specialists was significantly higher, indicating that the community maintained considerable evolutionary plasticity and ecological resilience under low-disturbance conditions. Given that generalists are less affected by environmental fluctuations and specialists can exploit additional resources by occupying novel ecological niches, the contraction of generalists’ ecological niches limits their conversion to specialists, whereas specialists increase their likelihood of becoming generalists by expanding their niches (Bono et al., 2015; Yang Y. et al., 2023). However, this dynamic equilibrium was disrupted following drainage, as evidenced by an overall decline in reversal rates, reduced formation, diversification, and extinction rates among generalists, and contrasting trends observed in specialists. Notably, in the DTHB-DTHC region, specialists exhibited a rapid turnover characterized by high formation and extinction rates, reflecting short-term adaptive responses through rapid trial-and-error under intense environmental pressure. Specialists, being more susceptible to environmental resource limitations than generalists, are consequently more vulnerable to disturbances such as spirits drainage and habitat alteration, resulting in elevated extinction rates (Shi et al., 2025b). The increased speciation rate observed in generalists may be attributed to their exposure to diverse environments, each imposing distinct genetic requirements and evolutionary pressures (Sriswasdi et al., 2017). Conversely, specialists may exhibit lower speciation rates due to their restricted dispersal capabilities, which stem from their narrower ecological niches (Buckling et al., 2003). Across multiple sites, generalists tend to display higher diversification rates than specialists, potentially because generalist populations encounter a broader range of abiotic stressors (e.g., organic contaminants) and biotic interactions (e.g., competition), suggesting that evolutionary potential is influenced by both abiotic and biotic factors (Škaloud et al., 2019; Yang Y. et al., 2023). Additionally, consistent with previous research (Shi et al., 2025a), our findings showed that specialists exhibited higher diversification rates than generalists in the DTHB–DTHC reaches, suggesting that environmental heterogeneity and geographical differences induced by distillery drainage provide specialists with more locally adaptable sub-niches; however, the specific underlying mechanisms warrant further confirmation.

Generalists and specialists demonstrate distinct evolutionary potentials and environmental adaptations (Shi et al., 2025a; Yan et al., 2022). Our findings indicated that specialists exhibited greater environmental adaptation in areas impacted by spirits drainage, whereas generalists displayed higher environmental adaptation than specialists when the influence of spirits drainage was reduced. This contrasts with previous studies reporting that generalists possess superior environmental adaptation compared to specialists (Yan et al., 2022; Yang Y. et al., 2023). Specialists, characterized by narrow ecological niches and specific environmental requirements, showed enhanced adaptation in regions affected by spirits drainage. Conversely, generalists, which occupied broader ecological niches and exhibited greater resilience to environmental fluctuations, demonstrated higher adaptation than specialists in areas more distant from spirits drainage where environmental conditions were more heterogeneous (Chen et al., 2021; Rain-Franco et al., 2022; Ren et al., 2024). Collectively, this study elucidated how environmental stress induced by spirits drainage reshapes community evolutionary trajectories, driving ecosystem transitions from broadly adapted generalists to highly specialized taxa through a reversal of generalist-specialist dynamics. Importantly, these findings not only advance the understanding of the generalist-specialist trade-off theory but also provide valuable insights for ecosystem management and restoration amid global environmental change. In low-disturbance settings, prioritizing the maintenance and promotion of generalists may enhance system resilience, whereas in environments subject to intense disturbances, improving habitat suitability and extending the survival window of specialists may be critical for sustaining community structure and ecological functions.

### 4.3 Stochastic processes having a greater impact on community assembly

A combined analysis employing null and neutral models demonstrated that stochastic processes predominantly govern the community assembly of both generalist and specialist; however, notable differences exist in the relative influence of these processes, consistent with findings from studies conducted in dredged lakes and urban aquatic environments (Al et al., 2022; Yang Y. et al., 2023). Conversely, research on the Chishui River reported a stronger role for deterministic processes in shaping bacterial community assembly (Wu et al., 2022). Microbial community assembly is modulated by both deterministic and stochastic mechanisms, wherein deterministic processes affect microbial community fitness, while stochastic processes unpredictably modify community structure by generating independent species assemblages (Al et al., 2022). The equilibrium between microbial communities shaped by stochastic and deterministic ecological processes is substantially affected by environmental variables, geographic location, and other factors (Wu et al., 2023; Yang Y. et al., 2023). For instance, deterministic processes predominate in microbial communities of mountainous rivers, whereas stochastic processes, such as dispersal limitation, may be intensified in plains or transitional terrains due to variations in hydrologic connectivity (Shi et al., 2025b). Across all studied reaches, stochastic processes contributed significantly more to community assembly than deterministic processes, with generalist communities exhibiting greater dispersal capacity and reliance on drift. This observation suggested that, under the complex environmental gradients induced by distillery effluent discharge, generalist communities tended to preserve their structure and function through neutral processes, and their dynamic nature enabled rapid adjustment of community composition via dispersal and stochastic drift in response to environmental perturbations. This characteristic accounts for the significant correlations observed between generalist communities and various environmental factors (EC, TDS, SAL, As, Ni, Fe, etc.), i.e., the interaction between stochasticity and environmental variables drove structural changes within generalist communities.

In contrast, specialists exhibited lower mobility, and their community structure was not significantly correlated with most environmental variables, demonstrating only a limited response to DO and certain individual metal ions (e.g., Ni). This finding suggested that specialists had a diminished response to environmental filtering and were more influenced by spatial constraints, such as the distance from the outfall. Furthermore, the randomness process dominated community construction and contributed more to the construction of generalist communities than to specialists, which contrasted with several previous studies that have reported a stronger role of stochasticity in generalist community assembly (Al et al., 2022; Yang Y. et al., 2023). Nonetheless, some studies have also highlighted the predominant influence of stochastic processes on generalists (Yan et al., 2022). Habitat characteristics, topographic variation, and environmental conditions have been shown to enhance the impact of stochastic processes on the assembly of specialist communities (Shi et al., 2025a). The presence of distillery effluent increased the contribution of non-selective processes in the assembly of both generalists and specialists, with ecological drift identified as the primary driver of community assembly under such disturbance. This effect was likely attributable to ecological drift induced by the disturbance from distillery effluent. Overall, spirits effluent not only altered the evolutionary and adaptive trajectories of generalists and specialists within the river but also restructured their community assembly mechanisms: generalists displayed high stochasticity and broad environmental sensitivity in response to intense disturbances, whereas specialists were more constrained by spatial factors and contingent events. Finally, we emphasize that niche breadth analysis based on a single dataset may lead to classification results that are sensitive to data fluctuations. If data from different seasons or regions is used, the classification results may vary. Furthermore, due to the absence of sampling points unaffected by any human activities, the observed differences in existing results may partially reflect natural spatial heterogeneity rather than wastewater effects. It is worth conducting more in-depth research in the future.

## 5 Conclusion

This study investigated the distribution patterns, diversity, and community assembly mechanisms of generalists and specialists in the Chishui River under the influence of distillery effluent. Utilizing water quality data, DNA sequencing datasets, and comprehensive statistical analyses, we systematically elucidated the spatial distribution, evolutionary potential, environmental adaptations, and ecological assembly processes of these bacterial groups. This research represented the first comprehensive examination of the impact of distillery drainage on riverine generalist and specialist bacterial communities. The findings demonstrated that spirits effluent induced persistent community differentiation between generalists and specialists (Generalists and specialists accounting for 15.17 and 8.33% at DTHA, 12.81 and 11.37% at DTHB, 5.73 and 4.09% at DTHC, and 10.72 and 7.80% at DTHD). These results advance the understanding of microbial community structure and ecological dynamics within riverine ecosystems. Furthermore, the study underscores the necessity for enhanced long-term monitoring and management of spirits effluent discharge to facilitate the restoration of water quality and ecological integrity in the Chishui River. The outcomes also provide valuable insights to inform governmental policy development for water environment protection within the basin. Given the critical role of bacterial generalists and specialists in maintaining ecological functions in disturbed aquatic systems, future research will extend to additional river sections impacted by distillery effluent to validate and expand upon these findings.

## Data Availability

The relevant water quality and microbiological data are stored in the Figshare database, doi: 10.6084/m9.figshare.30932471. Additional data supporting the conclusions of this study is available in the article and Supplementary materials.
