# Supplementary material for: Distribution patterns and community assembly processes of generalists and specialists in river affected by wastewater discharge from distilleries in typical Chinese spirits production area in China
Source: Front Microbiol. 2026 Jul 9;17:1860769. doi: 10.3389/fmicb.2026.1860769 (PMC13393092; doi:10.3389/fmicb.2026.1860769)
Supplement: Supplementary file 1 [file Data_Sheet_1.pdf]

Supplementary materials

Distribution patterns and community assembly processes of generalists and specialists in river affected by wastewater discharge from distilleries in typical Chinese spirits production area in China

Lixi Deng[a,1], Ying Huang[b,1], Qihong Zhong[b], Yu Mu[a], Dong Li[a], Yaxi Liu[c], Hong Zhang[a], Chi Zhao[a], Shirui Yu[a], Fu Jiao[d,*], Xiuyuan Yang[e,f,*]

[a] School of Food Engineering, Moutai Institute, Renhuai 564507, China

[b] School of Brewing Engineering, Moutai Institute, Renhuai 564507, China

[c] School of Resources and Environment, Moutai Institute, Renhuai 564507, China

[d] Kweichow Moutai Co. Ltd., Renhuai 564501, China

[e] Guizhou Institute of Biology, Guizhou Academy of Sciences, Guiyang 550009, China

[f] Fanjing Mountain Ecological Station of Guizhou Institute of Biology, Guizhou Academy of Sciences, Tongren 554499, China

[1] This is the co-first author of the manuscript.

*Corresponding Author:

* Kweichow Moutai Co. Ltd., Renhuai 564501, China; Email: 188216015@qq.com.

*Xiuyuan Yang, Guizhou Institute of Biology, Guizhou Academy of Sciences, Guiyang 550009, China; Email: yangxy0599@163.com.

1.1 Division of sampling points

Of the 20 sampling locations, three were situated upstream of the discharge point (Q1–Q3), one at the discharge point itself, and sixteen downstream (H1–H40). Sampling sites were grouped as follows: DTQ (river section upstream of the distillery discharge point, approximately 3 km upstream, 3 sampling points), DT (distillery discharge point, 1 sampling point), DTHA (5 km downstream, 5 sampling points), DTHB (10 km downstream, 5 sampling points), DTHC (20 km downstream, 4 sampling points), and DTHD (40 km downstream, 2 sampling points).

1.2 Specific division of community assembly null model

Specifically, values of $|\beta NTI| \leq 2$ indicate stochastic assembly processes, whereas $|\beta NTI| > 2$ signify deterministic processes, with $|\beta NTI| \leq -2$ corresponding to homogeneous selection and $|\beta NTI| > 2$ to heterogeneous selection. The Raup-Crick index based on Bray-Curtis dissimilarity (RCbray) further differentiates assembly mechanisms: RCbray < -0.95 indicates homogenizing dispersal, RCbray > 0.95 indicates dispersal limitation, and intermediate values represent non-homogeneous dispersal.

1.3 PCR conditions

The reaction mixture includes: Mg^{++}, Q5 Reaction Buffer, dNTPs, Q5 High-Fidelity DNA Polymerase, 5X Q5 Reaction Buffer. DNA quality assessment: Agarose gel electrophoresis was used to detect the integrity of DNA samples, to evaluate the DNA quality. The temperature and number of cycles for PCR are shown in Figure S13.

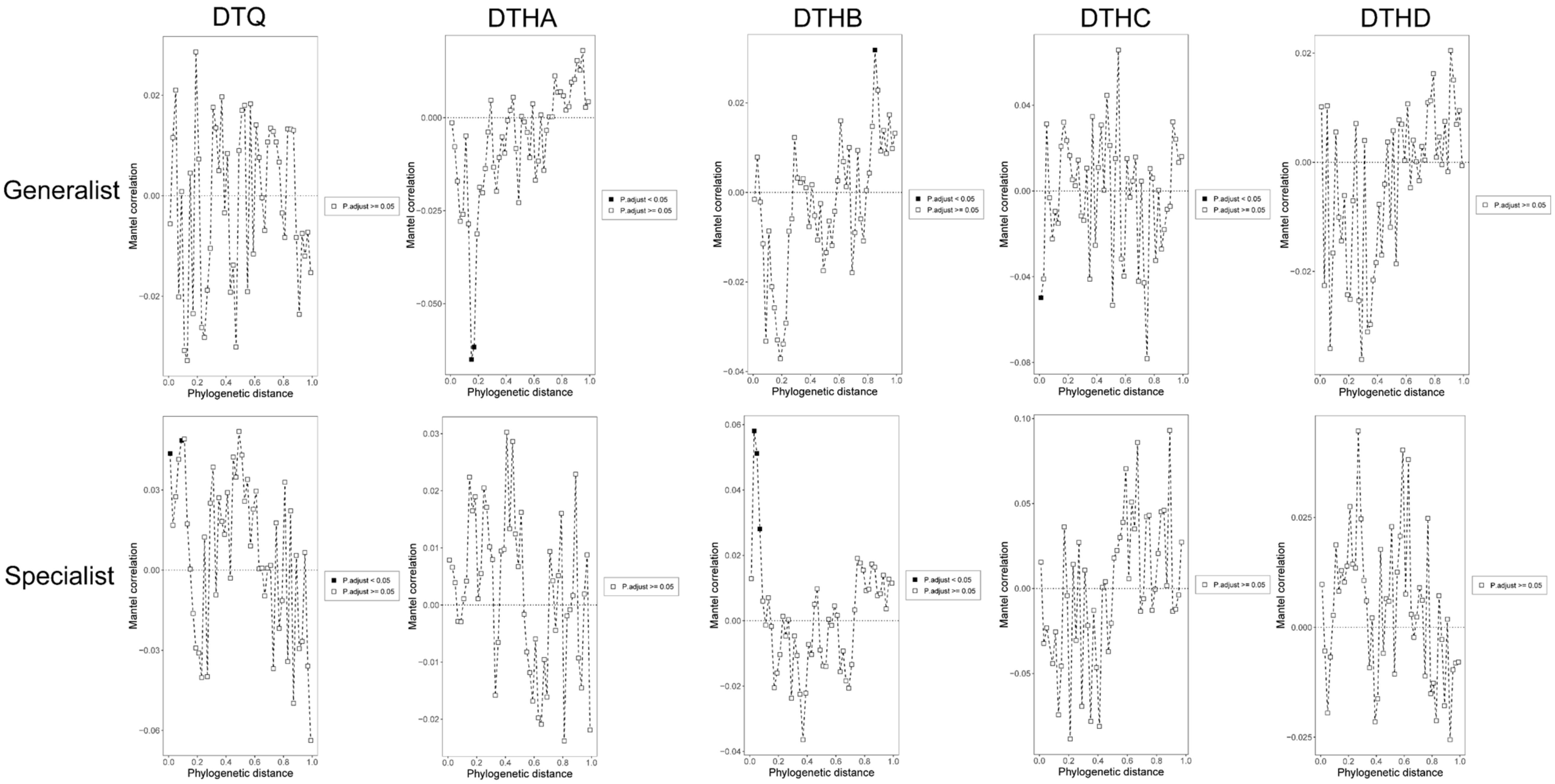

Figure S1 Phylogenetic signals of generalist and specialist communities in different river reaches

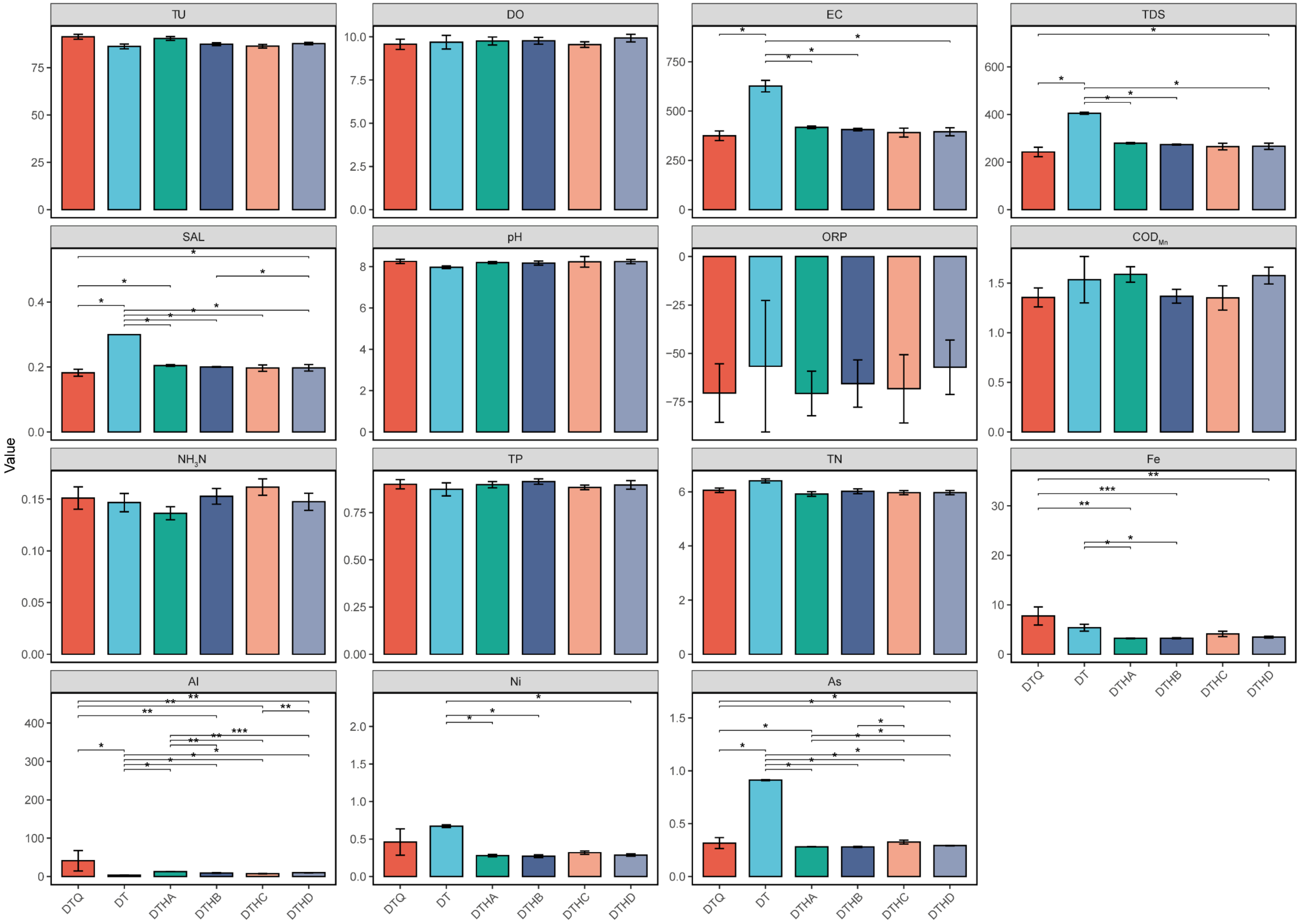

Figure S2 Water quality indexes in different river sections

Table S1 Water quality indicators of water discharged from distilleries into river

	Turbidity	DO	EC	TDS	SAL	pH	ORP	COD_{Mn}	NH_3-N	TP	TN	Fe	Al	Ni	As
Q3	90.01	9.84	301.40	186.92	0.15	8.14	-51.97	1.37	0.16	0.91	6.04	5.69	15.20	0.31	0.26
Q2	93.36	9.74	410.93	272.35	0.20	8.38	-60.53	1.23	0.16	0.90	5.99	13.87	97.43	0.81	0.42
Q1	90.73	9.12	410.53	268.45	0.20	8.24	-98.93	1.47	0.13	0.89	6.14	3.68	10.87	0.26	0.27
A0	86.24	9.69	626.33	405.17	0.30	7.97	-56.63	1.53	0.15	0.87	6.40	5.36	3.73	0.67	0.91
H1	92.52	9.93	423.93	286.00	0.21	8.14	-66.57	1.57	0.12	0.96	5.88	3.02	12.03	0.28	0.28
H2	90.28	9.02	419.37	274.95	0.20	8.06	-94.03	1.40	0.13	0.88	6.10	3.21	13.93	0.30	0.28
H3	93.14	9.92	433.73	290.55	0.21	8.23	-69.63	1.63	0.16	0.84	5.82	3.29	13.87	0.34	0.28
H4	90.28	9.36	411.60	273.00	0.20	8.25	-94.55	1.50	0.13	0.94	5.76	3.35	12.27	0.23	0.28
H5	86.08	10.55	399.60	273.87	0.20	8.30	-28.70	1.83	0.15	0.88	6.03	3.21	13.60	0.25	0.29
H6	86.48	9.48	413.17	277.12	0.20	8.26	-96.37	1.60	0.18	0.92	5.95	3.15	12.30	0.39	0.29
H7	85.78	10.76	399.87	273.45	0.20	8.22	-26.17	1.17	0.14	0.87	5.97	3.56	7.77	0.25	0.28
H8	90.74	9.35	398.63	269.75	0.20	8.22	-88.77	1.30	0.16	0.89	6.25	3.19	7.80	0.26	0.28
H9	87.54	10.08	394.63	272.86	0.20	8.49	-63.67	1.40	0.13	0.99	6.09	3.13	8.98	0.21	0.28
H10	86.52	9.18	424.10	275.17	0.20	7.68	-52.87	1.37	0.15	0.90	5.82	3.06	9.86	0.24	0.27
H15	86.93	9.57	372.57	257.01	0.19	8.33	-85.07	1.43	0.17	0.87	5.85	3.47	8.06	0.36	0.37
H20	85.84	9.53	408.90	273.37	0.20	8.14	-51.47	1.27	0.15	0.89	6.09	4.75	7.17	0.27	0.29
H25	87.31	10.33	419.33	281.88	0.21	7.95	-17.47	1.50	0.15	0.95	6.05	3.73	9.01	0.28	0.30
H30	88.68	10.52	434.97	292.62	0.22	8.41	-32.53	1.67	0.13	0.81	5.92	3.85	10.06	0.23	0.29
H35	85.78	9.46	416.10	284.48	0.21	8.36	-91.20	1.53	0.14	0.91	6.23	3.21	10.50	0.30	0.30
H40	89.14	9.41	307.83	206.37	0.15	8.26	-87.37	1.60	0.17	0.91	5.68	3.09	10.47	0.34	0.29
Wastewater	82.77	9.41	1267.22	834.57	0.64	8.09	-78.83	1.64	0.16	0.92	6.00	17.67	8.28	1.50	0.59

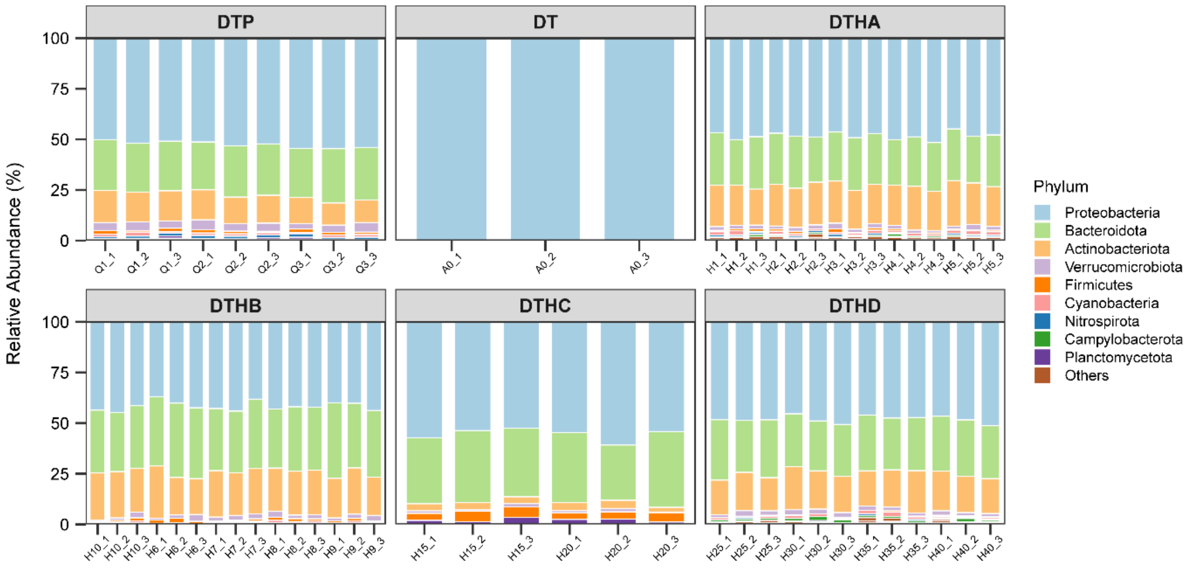

Figure S3 Species composition of generalist communities in different river reaches

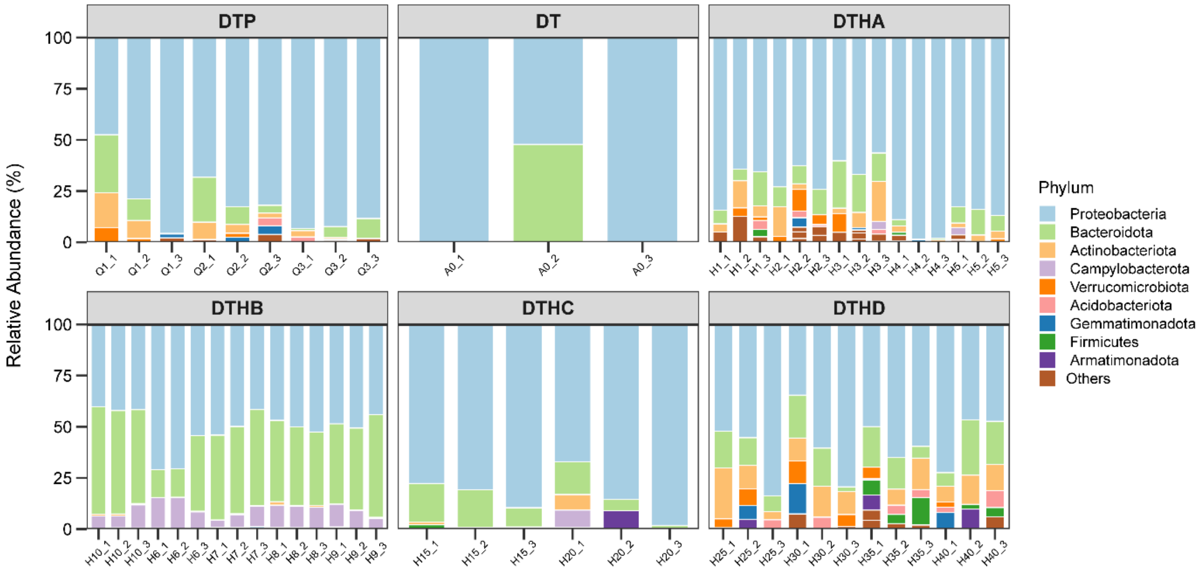

Figure S4 Species composition of secondary professional community in different river sections

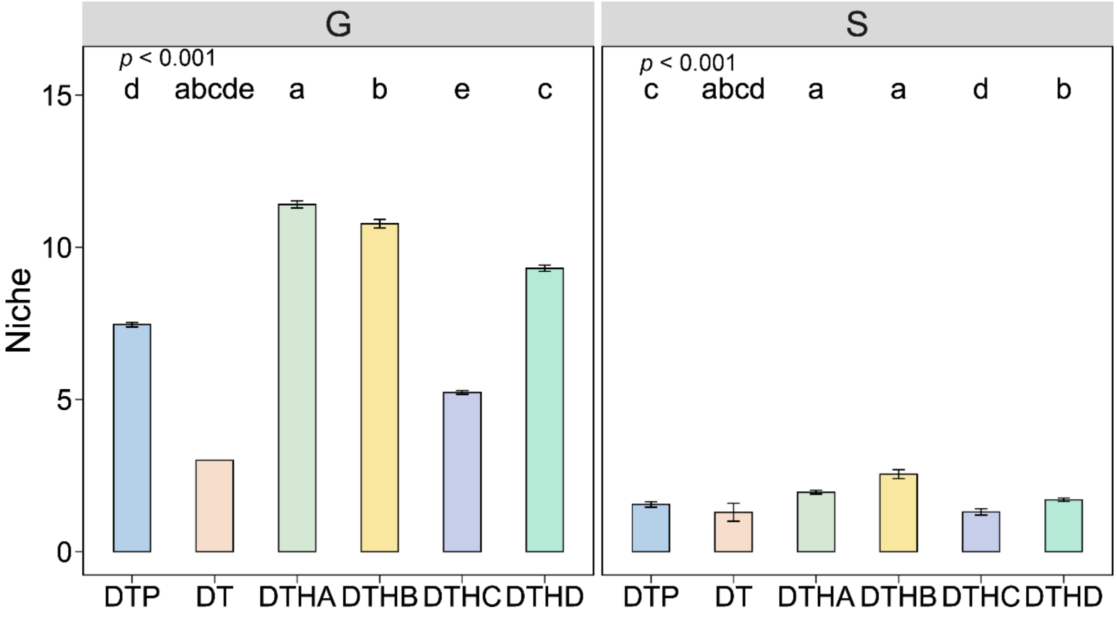

Figure S5 Niche breadth of generalist and specialist communities in different river reaches

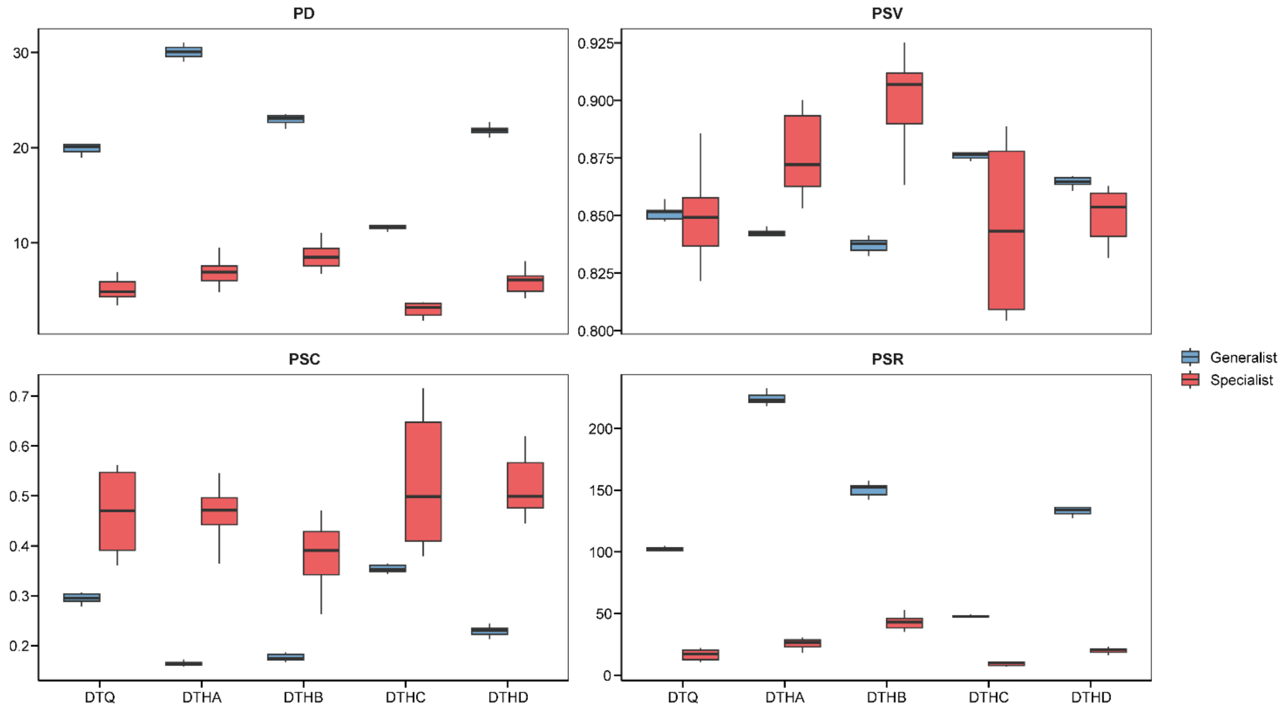

Figure S6 Phylogenetic diversity of generalist and specialist communities in different river reaches

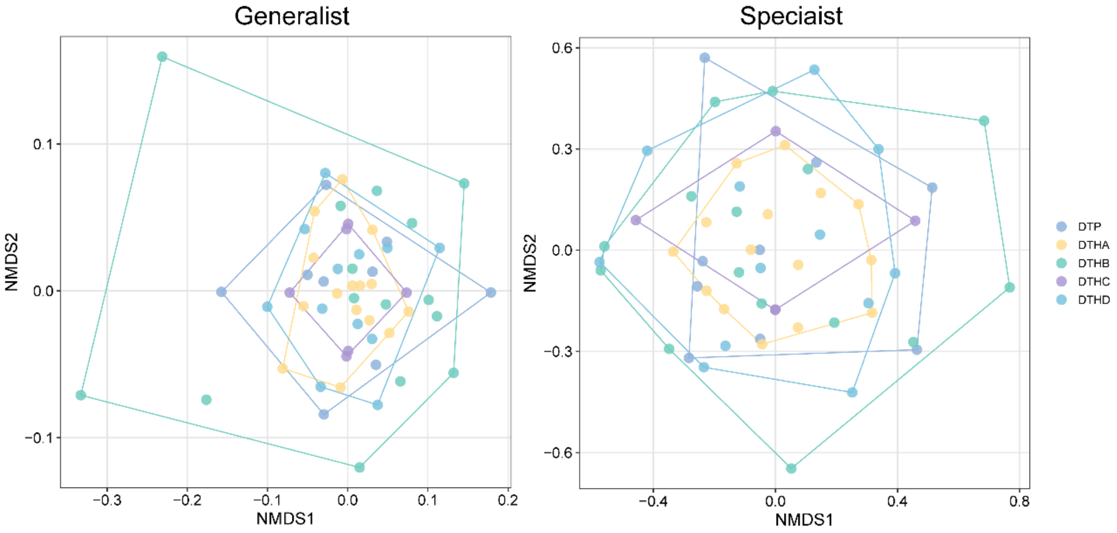

Figure S7 Non metric multidimensional scale analysis of generalist and specialist communities in different river reaches

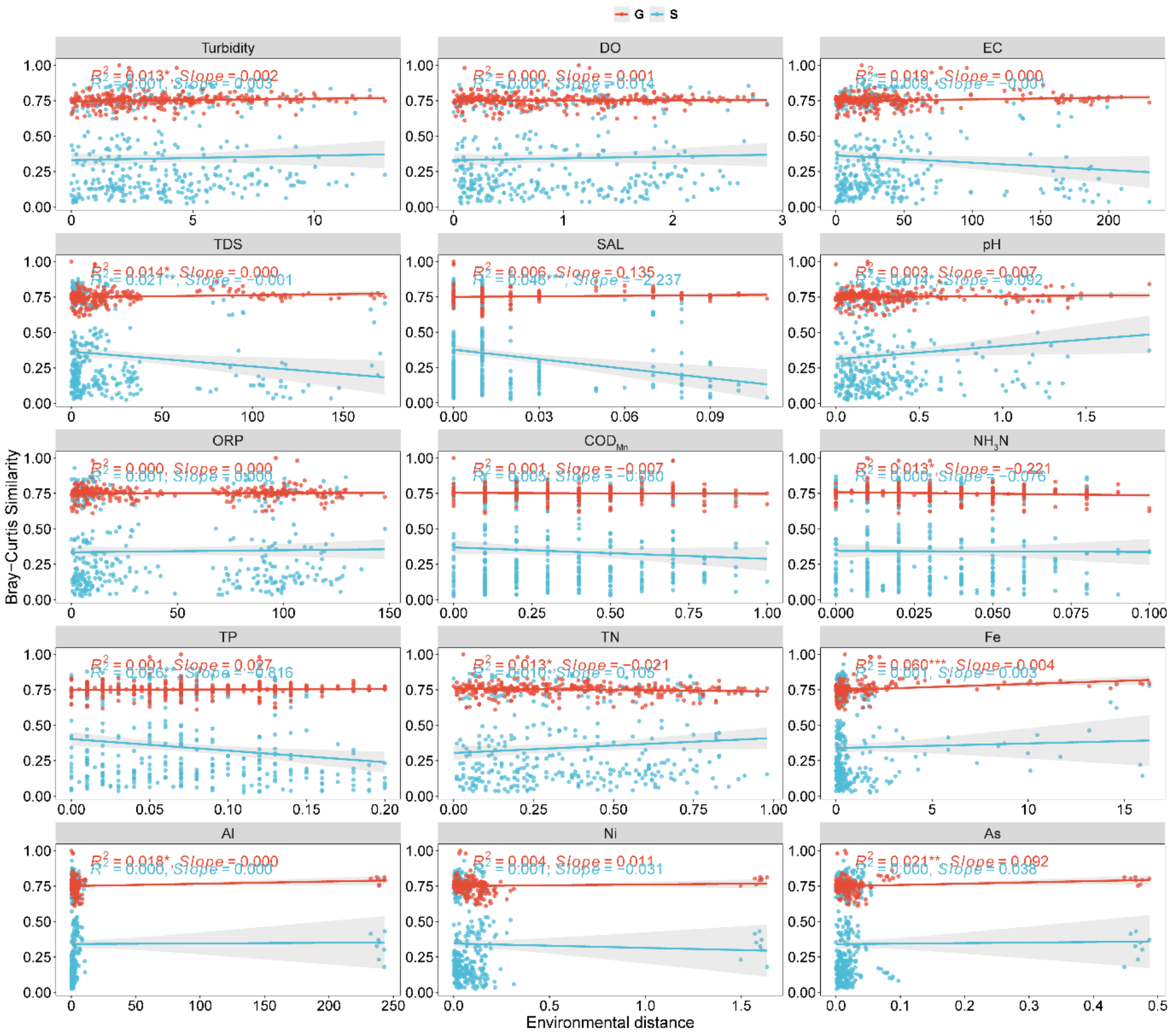

Figure S8 Relationship between Bray-Curtis dissimilarity and environmental distance.

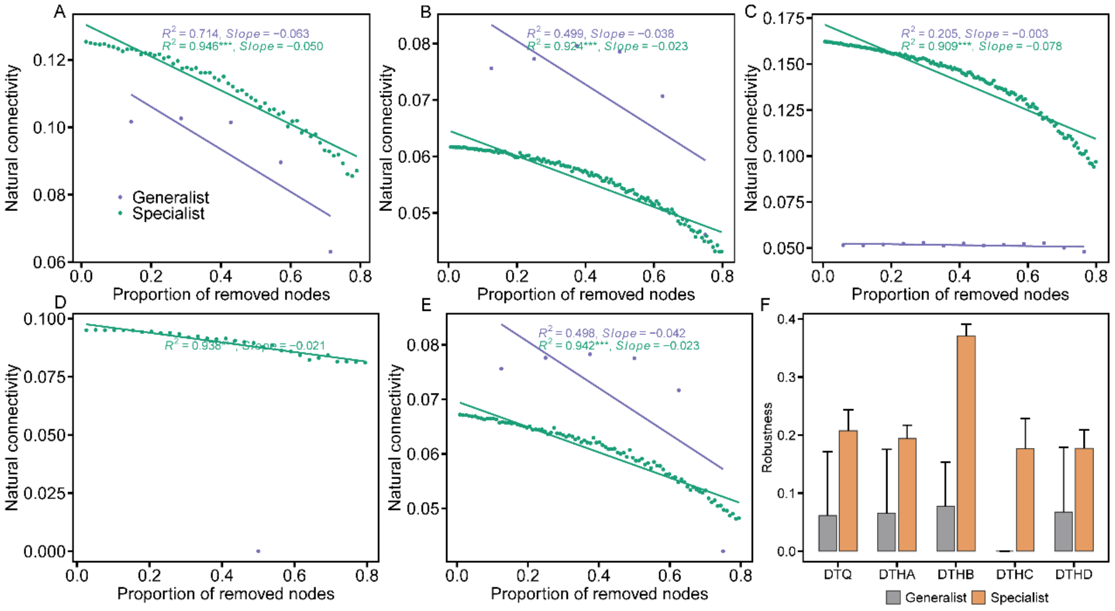

Figure S9 Natural connectivity (A–E) and network robustness (F) of microbial co-occurrence networks.

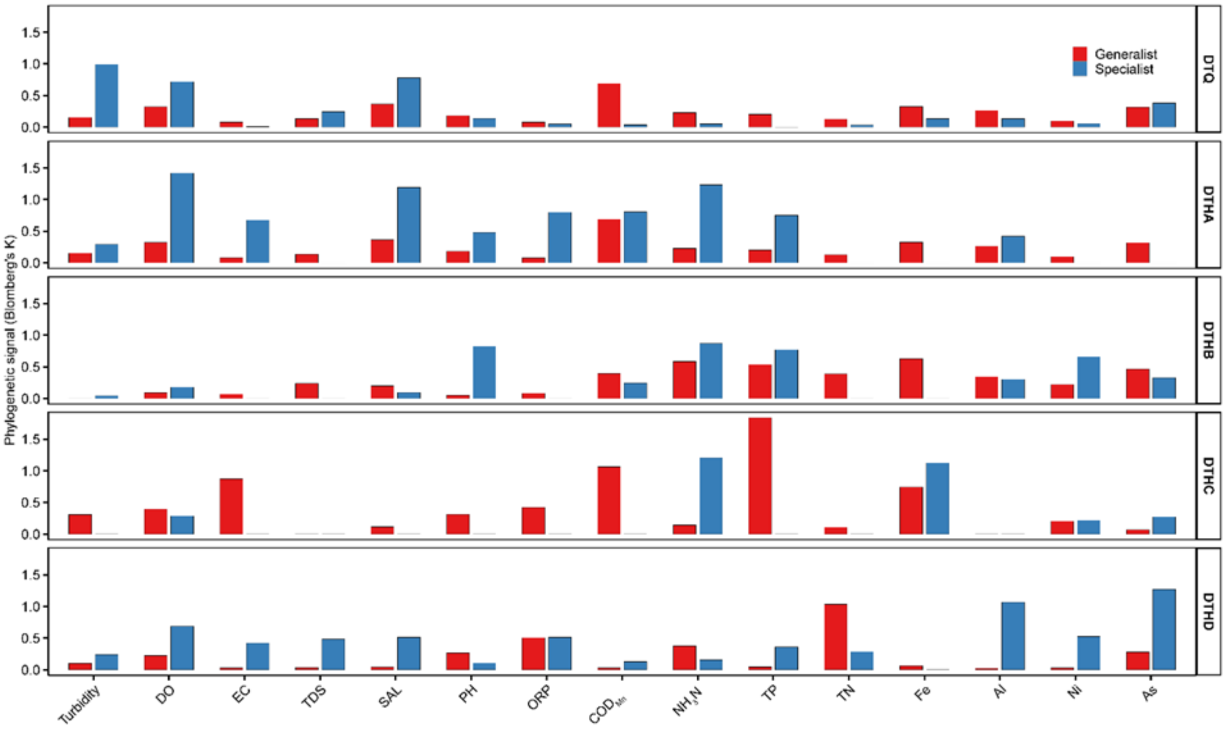

Figure S10 Environmental adaptation of generalists and specialists. Phylogenetic signals demonstrate the conserved nature of generalist and specialist environmental preferences.

Table S2 Topological characteristics of microbial symbiotic networks

	Generalist						
	Positive.cor_num	Negative.cor_num	Average_degree	Average_path_length	Network_diameter	Network_density	Degree_centralization
DTQ	2.00	2.00	1.14	1.14	1.93	0.19	0.14
DTHA	1.00	3.00	1.00	0.84	0.85	0.14	0.00
DTHB	5.00	7.00	1.41	0.95	1.65	0.09	0.10
DTHC	1.00	0.00	1.00	0.99	0.99	1.00	0.00
DTHD	1.00	3.00	1.00	0.88	0.90	0.14	0.00
	Specialist						
	Positive.cor_num	Negative.cor_num	Average_degree	Average_path_length	Network_diameter	Network_density	Degree_centralization
DTQ	316.00	0.00	8.32	1.05	1.88	0.11	0.09
DTHA	692.00	0.00	8.76	3.06	8.58	0.06	0.05
DTHB	1232.00	0.00	14.16	4.40	11.85	0.08	0.14
DTHC	90.00	0.00	4.62	1.60	4.76	0.12	0.06
DTHD	442.00	0.00	7.56	3.27	10.37	0.07	0.05

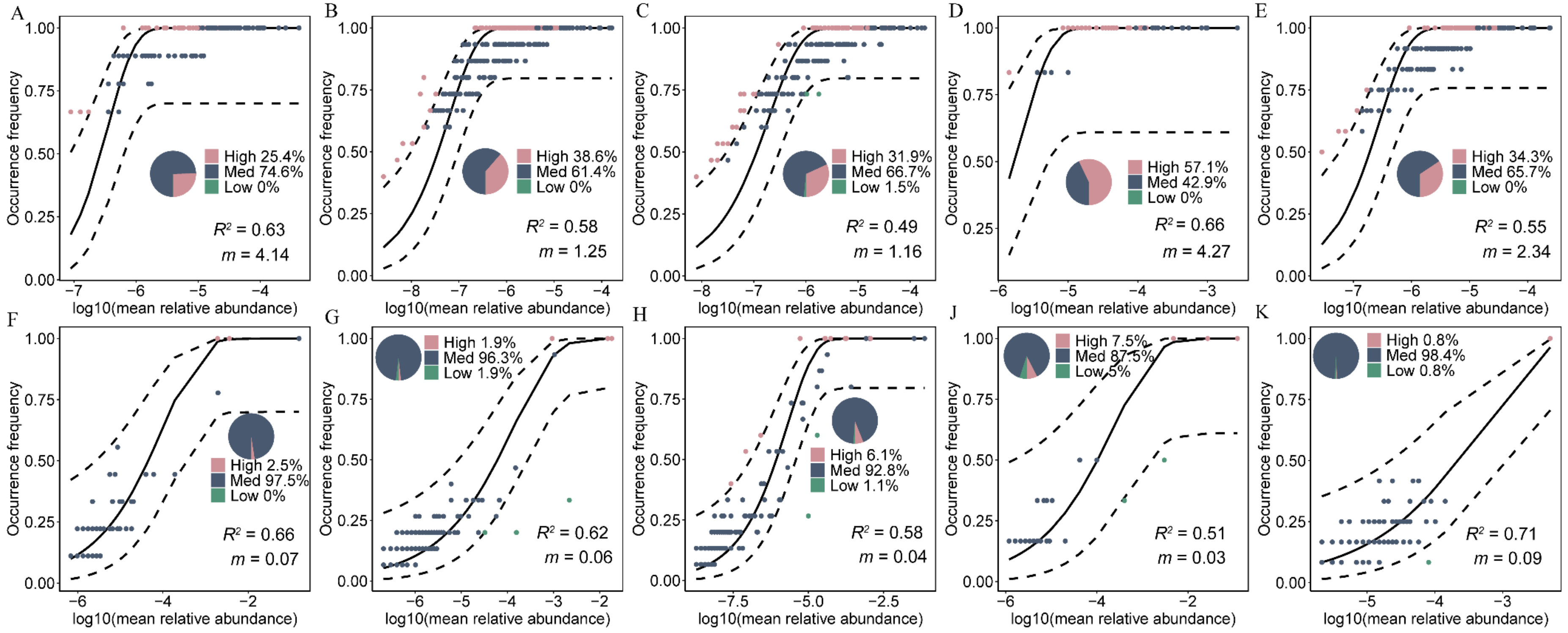

Figure S11 Fit of the neutral community model. The red and blue circles represented ASVs that occurred more and less frequently than predicted, respectively. The solid black line indicated the best fit to the neutral community model and the dashed black lines represented the 95% confidence intervals. m was the estimated migration rate and R2 was the fit to the neutral community model

Table S3 The relationship between βNTI and environmental factors

	Generalist_βNTI	Specialist_βNTI
Turbidity	-0.11	-0.20
DO	0.05	**0.29**
EC	0.04	-0.24
TDS	0.01	-0.17
SAL	0.05	-0.17
PH	-0.05	0.15
ORP	0.06	0.13
COD_{Mn}	0.05	0.09
NH_3N	0.10	0.13
TP	-0.16	-0.12
TN	-0.05	0.01
Fe	-0.21	-0.14
Al	-0.17	0.09
Ni	-0.14	0.11
As	-0.13	0.06

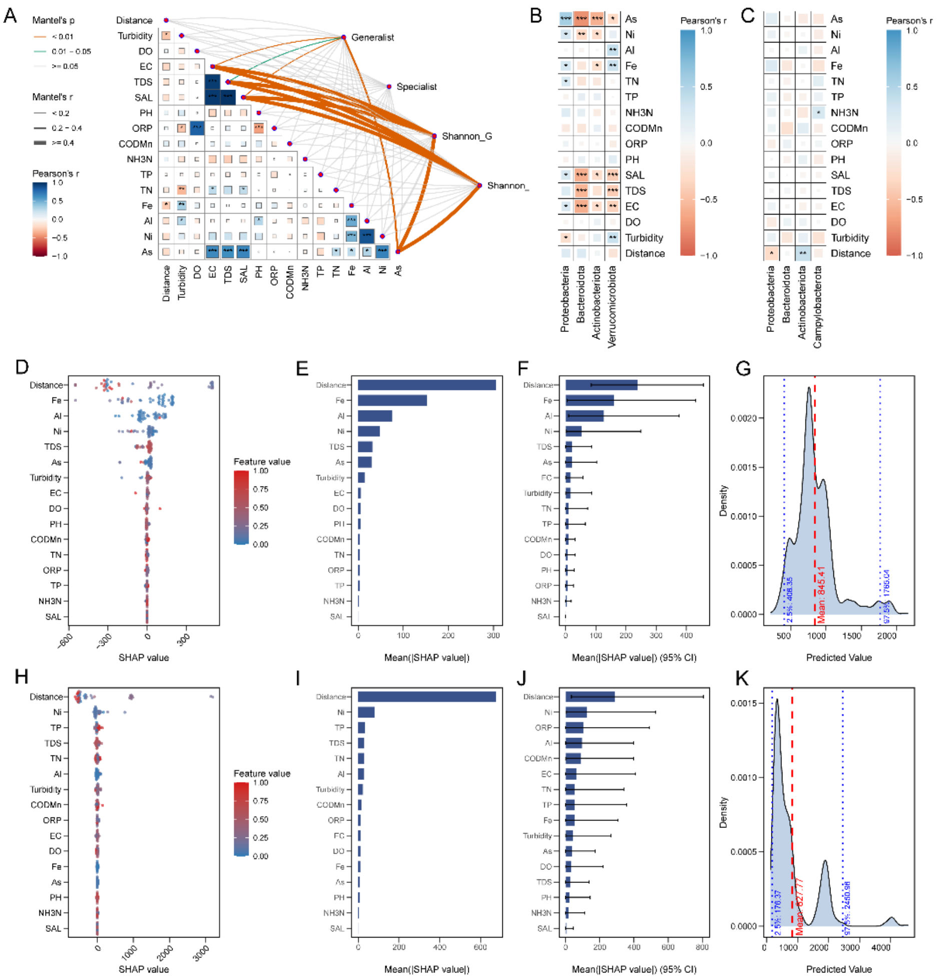

Figure S12 Environmental factors affecting generalist and specialist communities. (A) Environmental drivers of generalist and specialist communities and their diversity, as assessed by Mantel tests; (B) correlation between main contributing taxa of generalists and environmental factors; (C) correlation between main taxa of specialists and environmental factors; (D-G) machine learning-estimated environmental factor contributions to generalist communities could be interpreted, with D and E being the swarm and histogram, respectively, and F and G being the Monte Carlo estimated environmental factor contributions and their probability distributions; (H-K) could explain the machine learning estimated environmental factor contributions to specialist communities, H and I were swarm plots and histograms, respectively, and J and K were Monte Carlo estimated environmental factor contributions and their probability distributions, respectively. Explainable machine learning model parameters (generalists): R^2=0.99976; RMSE=9.3877; MSE=88.1296. Explainable machine learning model parameters (generalists): R^2=0.99991; RMSE= 10.8244; MSE=117.168.

	Bacteria (V3-V4)		Remark	500bp
338F	ACTCCTACGGGAGGCAGCA			
806R	GGACTACHVGGGTWTCTAAT			
Amplification conditions				
98°C	3 min			
98°C	30 s	}		26 cycles
52°C	30 s			
72°C	45 s			
72°C	5 min			
12°C	……			

Figure S13 PCR temperature and number of cycles
